# Three Cases of Pulmonic Inflammation, Illustrative of Observations on Peripneumony, Inserted in the Medical and Physical Journal for November, 1814

**Published:** 1815-03

**Authors:** W. Robertson


*For the Medical and Physical Journal.*

*Three Cases of Pulmonic Inflammation, illustrative of Observations on Peripneumony, inserted in the Medical and Physical Journal for November,* 1814. By W. Robertson, M.D. A.O.L. and C.C.E.

"Oportet itaque, ubi aliquid non respondit, non tanti putare authorem, quanti ægrum, et experiri aliud atque aliud."—Celsus.

"La veritê, et la raison sont communes á chacun, et ne sont pas plus a qui les a dites premierement, qu'à celui qui les a dites aprês."—Montaigne.

IN some of the diseases of the order Phlegmasiæ, the general depletory system, so universally and judiciously inculcated, seems in many instances to have little influence in subduing the morbidly increased action of the viscus inflamed, though assisted by the collateral and potent adjuvants of topical subtraction of blood, counter irritation of cantharides, and rigid observance of all the parts of the antiphlogistic regimen. Even when the general reaction of the system, occasioned by, or attendant on, the local inflammation, has been overcome by the means above enumerated,

 still still the disease of the particular viscus often proceeds to a fatal termination, with as much certainty and celerity as if no remedies had been exhibited. If this be true, which painful experience has, we have no doubt, taught every physician, it must be apparent, that the doctrine of the extreme vessels being possessed of an action *sui generis* independent of their parent source,* and not amenable to its laws, in some diseases at least, if not in health also, is consonant to some of the phenomena attendant on inflammation. Ought we not to a certain amount to adjust our prognosis, and regulate our curative measures, from a knowledge of this circumstance? The frequent occurrence of visceral adhesions, effusions, suppurations, and tumors, demonstrated on dissection, and which were not only undiscovered during life, but absolutely unattended by any fever or apparent reaction of the vascular system, seems to promise validity to these speculations. However this may be, the circumstance is indubitable that visceral inflammation often proceeds to a fatal termination independent of the depletory remedies of the physician. In consequence of disappointment and failure from the usual remedies, we have found it expedient to have recourse to other means to combat the secondary symptoms of visceral inflammation, whether as the concomitant or product of general phlogistic excitement, or, as the attendant or offspring of febrile pyrexia; and these, in very many instances, we have experienced to be adequate to the task, and commensurate with our most anxious feelings, though, doubtless, subject, like every human effort in the cause of mortality, to that uncertainty which, from the beginning, has been impressed on man himself.† We shall not enter here

* See Cullen's first lines, vol. I. page 270. See also, Morgagni, Epist. xxi. Boneti Sepulchret. tom. i. p. 508; and more particularly Diemenbroëk. Anatom. lib. ii. cap. 13, p. 308, as quoted by Van Swieten, vol. 8, p. 261. In which a case is related of the lungs being found after death, not only adhering to each side, "but likewise grew so firm to the mediastinum and diaphragm, that they could not be separated from thence without laceration, yet the man had *lived in health without any difficulty of respiration.*"

† I am unconscious of wishing to detract from the merit of others. Dr. Hamilton, of Lynn Regis, (in Duncan's Medical Commentaries) some years ago recommended similar remedies nearly to those which we now inculcate, though in different stages of the same diseases; and Dr. Yates, of London, has given an excellent paper on this subject in the Annals of Medicine, vol. ii. hist. 2d.

on on the theory of inflammation, whether it is to be considered as a state of increased action of the vessels, as maintained by most of the ancient and modern physicians down to a comparatively late era; an obstruction and lentor in the minute vessels;* a spastic stricture with inordinate action in these;† or whether it consist in a diminished action in the same vessels as adduced by some moderns.‡ This subject is, doubtless, worthy of rigid investigation, (and, on this head, we refer to the authors enumerated in the foot note;) but our object at present is more a practical one, "neque quærendum esse, quomodo spiremus; sed quid gravem tardumque spiritum expediat.§

In regard also to the order Febres, we are convinced, both from long and repeated observations, and from reiterated dissections, that local inflammations, congestions, and effusions in viscera most essential to life, have frequently existed where no such occurrences were suspected, and which were the immediate cause of the subsequent fatality; particularly in warm climates, where we have had frequent opportunities of witnessing such events. The late depletory system of treating these maladies, which has proved so efficient, bears me out in some degree in these assertions. But I hope to enter more fully, at some future period, on this subject, and to be able to elucidate certain practical points relative to the use of mercury and the proper period for its exhibition in the remittent fevers and some other diseases, as they appear in warm countries; a discussion which has not a little agitated, by the discrepancy of sentiments adduced, the medical world, and called into existence, a degree of animosity of feeling, and virulence of expression, which have by no means tended to enforce conviction, dissipate doubt, or induce unity of opinion. In regard to the modus operandi of mercury in the cure of the secondary stages of venereal inflammation, we have little to adduce; though this, in most instances, might readily be assigned, when mercury is considered as a direct stimulant, affecting every sensible and moving fibre, by the induction of permanent excitement of the living system; as an indirect and general evacuant, en-

---

* Vide Boerhaave and his commentators, Gaub. Pathol, 249, I.

† See Cullen's first lines, vol. i. p. 279.

‡ See a paper in the Edinburgh Medical and Surgical Journal, No. 15, 1765, in which Professor Berlinghien, of Pisa, has brought forward this theory. See a System of Surgery by Mr. Latta, vol. i. p. 99; also, a Treatise on Febrile Diseases, and an Essay on Fever, by Dr. Wilson, of Worcester.

§ Celsus de Med. Præf. p. 2.

tering tering into the circulating medium and pervading every part of the animal body, and finally as a powerful, though not generally acknowledged, tonic. I have only further to observe, that it appears to me, that the introduction of this metal into the system has a peculiar efficacy, in rendering the circulation uniform throughout the body, and, in consequence, in preventing and removing morbid determinations of blood to particular organs; while all this may be effected under due regulation, without much acceleration of the pulse. If this doctrine be well founded, mercury must unquestionably be considered as one of the most powerful agents in the physician's possession for combating the diseases we have been treating of.

Case I. March 2, 1814.—I was requested to visit Mr. ———, æt. 47, whom I found in the erect posture,* labouring under great difficulty of breathing, much aggravated by an incessant cough and pain between the 7th and 8th ribs, attended by a sense of weight, oppression, and increased heat in the whole of the right side, extending occasionally under the breast bone. The cough is accompanied with a copious expectoration of bloody sputa, excreted without much difficulty. Any attempt at a full inspiration, does not seem to aggravate the pain, but evidently induces such an alarming sense of suffocation, as to prohibit any considerable effort for the purpose, though his frequent abortive endeavours shew his solicitude to accomplish the full elevation of the ribs. He cannot lie on the affected side, reclining generally on his back when in bed, but feels no inclination till nearly exhausted from fatigue, to desist from the erect posture. The face is considerably swollen and puffy in its appearance, while the forehead is bedewed with an unctuous moisture. The eyes are heavy and denote much uneasiness, suspicion, and anxiety; the cheeks and lips strongly indicate the probable condition of the lungs, they are purple, inflated, and glazed; in some places, spotted with alternations of purple and a dirty yellow, while the neck, which he sedulously preserves uncovered, is altogether of a dingy ochreous hue. The frequent motions of the alæ nasi, which yet always appear distended, and the constant elevation of the points of the shoulders, conjoined to a rapid and anxious utterance, when interrogated, complete the external symptoms of a pectoral disease. His pulse is 108, regular, but feeble; tongue white, though moist towards the

* Vide Aretæus, de Causis et Signis Morb. Acut. p. 10, 11; et Hippocrates, in Prognosticis, p. 603.

edges, edges, and is tremulous when extended; urine deficient in quantity and high coloured; belly costive; appetite quite gone, and he says he frequently vomits the food he may have been induced to swallow. Thirst urgent; skin hot, but irregularly so, for on the head and neck there is a partial moisture, while the palms of the hands and soles of the feet are hot and dry. The body has suffered considerable emaciation.

These complaints commenced ten days ago, with alternations of cold and hot fits, frequent flushings of the face, and other symptoms of pyrexia; these were succeeded by pain in the side, cough, and difficulty of breathing; all of which he attributes to exposure to cold after being much heated. He was blooded on the third day of the disease, which produced some remission of his complaints, but they soon after recurred, and have gradually assumed their present aggravated form. Applicetur lateri quâ dolet, Emplastrum epispasticum satis amplum. Injiciatur Enema domesticum vesperi. Bibat decoctum hordei ad libitum, et sumat semunciam misturæ sequentis, ungenti tussi.

℞. Mucilaginis Acaciæ ℥iſs.
Aquæ Cinnamomi ℥v.
Syrupi Croci ℥i. Misce.

3d. Blister has risen well, had one motion from the enema. Pulse 104 and small, but regular. Much dyspnœa amounting almost to orthopnœa, and expectoration of viscid matter tinged with blood. Countenance very livid, and lips of a venous colour. Had much distressing cough in the night, and the head seems not quite clear this morning. Rep. Enema vesperi, si opus sit.

℞. Submuriatis Hydrargyri, Pulveris Antimonialis āā gr. iij. Opij gr. 1.
Conservæ Rosæ q. s. fiat Bolus.
Sumat statim et repetatur hora somni.
Fiat fonticulus ex parte vesicata ope unguenti Pulveris Cantharidum.
Continuatur Mistura Mucilaginosa & Habeat Vini Rubri ℥iv. Partitis vicibus sumendas.

4th. Dyspnœa and cough considerably relieved; skin moist, and heat moderate. Has had two stools; enema not given; pulse 100, soft and regular; tongue moist and cleaner; complains excessively of pain from the issue. Applicetur Cataplasma ex mica panis fonticulo. Repetatur Bolus, horâ somni. Continuentur Mistura & Vinum.

5th. Cough and breathing much easier, though the latter is far from being perfect. Pain of side cannot be distinguished from the pain of the issue, which, however, is better since the the application of the poultice. Head clear, and he talks with more composure; slept for some hours after taking the bolus, and awoke covered with perspiration. Pulse 96, soft and regular; much expectoration tinged with blood; countenance less livid, and the skin of the neck has in some degree lost its dirty hue. Injiciatur Enema domesticum vesperi. Continuentur Medicamenta.

6th. Breathing and cough and pain of side much relieved; a copious stool from the injections; face and lips much improved in colour; pulse 96; tongue moist, though not clean; heat moderate; urine of natural colour and rendered in greater quantity; expectoration still very copious. Omittatur bolus. Continuentur alia.

℞. Liquoris Ammoniæ Acetatis ℥iij.

Misturæ Camphoræ ℥xij.

Liquoris Antimonii Tartarisati gtt. xx.

Syrupi Croci ℥i. fiat haustus 4tis horis sumendus.

7th. Is much better in every respect; pulse 100; belly regular. Continuentur Medicamenta & Vinum.

8th. Has suffered much in the night from severe fits of coughing; expectoration much diminished; pulse 108; skin dry and communicates a sense of pungent heat to the hand. Continuentur Medicamenta, & hora septima vespertina, sumat Pulveris Ipecacuanhæ ℈i. pro emetico.

9th. Vomit operated well; skin in a perspirable state; much bloody sputum excreted. Intermittatur Vinum; continuentur alia. To have milk diet.

Note.—From this period till the 15th, when the patient was convalescent, the usual remedies were administered, and with the exception of occasional slight returns of febrile action, (dissipated by emetics and diaphoretics,) nothing worth noting occurred.

*Observations.*—This is evidently a case of Peripneumony and at the period of the Reporter's being consulted was too far advanced to admit of bleeding; for, when any effusion has taken place, (and this event, to a certain extent, more frequently occurs, I suspect, in the low passive inflammation of peripneumony, than is generally imagined,) venesection debilitates the patient without procuring any alleviation of the symptoms, at least any alleviation commensurate with the debility induced. Moreover, when expectoration has taken place, and which is often critical, it is very generally repressed by the detraction of blood. Blisters in these cases are most estimable remedies, and are adapted both to the conditions of inflammation and effusion; they seem to operate not only by derivation, but by increasing the action of one one set of vessels, and thus producing a diminution in that of others, and besides they are powerful antispasmodics. But at this period of the disease, I consider the introduction of mercury into the system, combined with opium, and occasionally antimony, as the most certain mode of cure we are acquainted with. It is particularly to be noted, however, (as happened in this case,) that the sudden subtraction of the mercurial treatment almost certainly induces a relapse or renewal of the dangerous symptoms, a circumstance I shall have occasion more particularly to illustrate in a subsequent case.* By referring to the report of the 8th, it will be seen, that very considerable febrile excitement, with an increase of heat and dryness of skin, occurred subsequently to the abstraction of the mercurial treatment, and this augmentation of temperature was probably attended by so considerable a spasm on the capillaries of the surface as to preclude the excretion of perspirable matter, and, in consequence, to aggravate the disease by repressing the determination from the centre.† After the discontinuance of the mercury in this case, it appeared advisable to produce a farther determination from the viscera, by means of diaphoretics, which in this instance was successful; but they cannot always be depended on, and are highly debilitating. An emetic very generally affords relief, when the expectoration from any cause is suddenly repressed, and was accordingly administered in this case with much advantage. Had, however, the mercurial treatment been continued, I feel little hesitation in asserting, that the disease would have been subdued in a much shorter period, the danger from effusion decreased if not totally dissipated, and an uniform action throughout the system permanently established. Though I conceive that this point will readily be conceded, yet it must be acknowledged, that in pulmonic diseases attended with little danger, the advantages which are likely to accrue from the mercurial action, will not be commensurate with the inconveniences which are to result from, and are necessarily attendant on, the induction of a permanent excitement from this remedy, and that in these cases, therefore, the usual means should be confided in, as

---

* See a case detailed by Dr. Yates, formerly of Bedford, now of London, Annals of Medicine for 1802, p. 397.

† It is well known, when the heat of the body rises above a certain point, (say 105°) that the capillaries of the surface are powerfully constricted; and, on the contrary, when the reaction is moderate, the orifices of the skin often relax and carry off the febrile heat by perspiration. See Dr. Currie's Medical Reports, p. 160.

adequate to the removal of the disease; but in such lamentable cases as I am about to detail, the exhibition of mercury and opium, with the occasional addition of antimony, and the free use of diffusible stimuli appear to be the most estimable remedies in the possession of the physician, and the most likely to deliver the oppressed and toil-worn patient from the dominion of an almost intractable disease.

The two following cases will be given in the next Number.

*Berwick-on-Tweed,* W. ROBERTSON.

*Dec.* 28, 1814.

---

*For the Medical and Physical Journal.*

